# Hexachlorobenzene as a differential modulator of the conventional and metronomic chemotherapy response in triple negative breast cancer cells

**DOI:** 10.37349/etat.2024.00218

**Published:** 2024-03-21

**Authors:** Yamila Sanchez, Mariana Abigail Vasquez Callejas, Noelia Victoria Miret, Gabino Rolandelli, Catalina Costas, Andrea Silvana Randi, Alejandro Español

**Affiliations:** Institute of Experimental Endocrinology and Oncology “G. Salvatore”-National Research Council (IEOS-CNR), Italy; ^1^Center of Pharmacological and Botanical Studies (CEFYBO)-National Council for Science and Technology (CONICET)-University of Buenos Aires, Buenos Aires C1121ABG, Argentina; ^2^Laboratory of Biological Effects of Environmental Pollutants, Department of Human Biochemistry, School of Medicine, University of Buenos Aires, Buenos Aires C1121ABG, Argentina; ^3^Department of Pharmacology, School of Medicine, University of Buenos Aires, Buenos Aires C1121ABG, Argentina

**Keywords:** Paclitaxel chemotherapy, metronomic chemotherapy, hexachlorobenzene exposure, triple negative breast cancer

## Abstract

**Aim:**

Triple negative breast cancer (TNBC) is usually treated with high doses of paclitaxel, whose effectiveness may be modulated by the action of environmental contaminants such as hexachlorobenzene. High doses of paclitaxel cause adverse effects such as low cellular selectivity and the generation of resistance to treatment due to an increase in the expression of multidrug resistance proteins (MRPs). These effects can be reduced using a metronomic administration scheme with low doses. This study aimed to investigate whether hexachlorobenzene modulates the response of cells to conventional chemotherapy with paclitaxel or metronomic chemotherapy with paclitaxel plus carbachol, as well as to study the participation of the MRP ATP-binding cassette transporter G2 (ABCG2) in human TNBC MDA-MB231 cells.

**Methods:**

Cells were treated with hexachlorobenzene alone or in combination with conventional or metronomic chemotherapies. The effects of treatments on cell viability were determined by the 3-(4,5-dimethylthiazol-2-yl)-2,5-diphenyltetrazolium bromide assay and the nuclear factor kappa B pathway participation was evaluated using a selective inhibitor. ABCG2 expression and its modulation were determined by western blot.

**Results:**

Results confirmed that paclitaxel reduces MDA-MB231 cell viability in a concentration-dependent manner. Results also showed that both conventional and metronomic chemotherapies reduced cell viability with similar efficacy. Although hexachlorobenzene did not modify cell viability *per se*, it did reverse the effect induced by the conventional chemotherapy, without affecting the efficacy of the metronomic chemotherapy. Additionally, a differential modulation of ABCG2 expression was determined, mediated by the nuclear factor kappa B pathway, which was directly related to the modulation of cell sensitivity to another cycle of paclitaxel treatment.

**Conclusions:**

The findings indicate that, in human TNBC MDA-MB231 cells, in the presence of hexachlorobenzene, the metronomic combination of paclitaxel plus carbachol is more effective in affecting the tumor biology than the conventional therapeutic administration scheme of paclitaxel.

## Introduction

Cancer is a significant cause of morbidity and mortality worldwide. In 2020, almost 20 million new cases with 10 million deaths were recorded [1]. In particular, breast cancer is the most frequent in women worldwide, with 2,261,419 new cases and 684,996 deaths in 2020. Thus, in women, breast cancer constitutes the leading cause of death linked to malignant diseases [2], with a forecasted increase of 40.8% in its incidence and of 51.9% in its mortality by 2040 [3]. Breast cancer is a heterogeneous disease and four main subtypes of tumors have been described according to their genetic profile. The subtype with the worst prognosis is triple negative breast cancer [TNBC; estrogen receptors-/progesterone receptors-/human epidermal growth factor receptor 2 (HER2)-], due to its high probability to metastasize and the lack of a specific target for its treatment [4].

In general, breast cancer treatment includes surgery, radiotherapy, endocrine therapy, immunotherapy and chemotherapy. One of the most widely used chemotherapeutics in the treatment of breast cancer is the cytostatic drug paclitaxel (PX), which acts by inhibiting the depolymerization of microtubules and in turn arresting cell cycle. Due to its mechanism of action, it is not tumor tissue-specific. Classically, it is administered in a single dose, the highest possible, called the maximum tolerable dose, which concomitantly causes high toxicity, requiring long intervals between successive treatment cycles to allow the patient to recover from the undesirable side effects. This administration scheme can also favor the appearance of resistance to treatment mediated by the increased expression of ATP-binding cassette transporter G2 (ABCG2) proteins [5]. Although in breast cancer the resistance signaling pathways induced by PX remains unclear, it is known that it can induce the activation of the nuclear factor kappa B (NF-κB) pathway [6], and this can in turn regulate the expression of genes related to multidrug resistance proteins (MRPs) like ABCG2, which actively transport PX to the extracellular space, inhibiting its action [7]. Drug resistance is responsible for up to 90% of deaths in breast cancer [8].

To avoid these adverse effects, metronomic therapy (MT), which is based on the administration of much lower drug doses than the maximum tolerable dose, with short drug-free intervals, arises as a potential alternative strategy. This strategy increases the effectiveness, reduces side effects and induces other concomitant responses that help to eradicate the tumor [9].

MT is usually linked to the introduction of repurposing drugs, which are compounds currently used in the treatment of other diseases and that have been found to exert antitumor actions. The benefit of using these drugs is that they have known pharmacokinetics/dynamics and side effects, and that, in most of the cases, they are available at low cost. In MT schedules, chemotherapeutic drugs may be administered in combination with others, as repurposing drugs, which have a synergistic effect [10].

In this context, previous research has determined that the treatment of breast cancer cells with a metronomic scheme based on the combination of low doses of PX and the muscarinic cholinergic agonist carbachol (Carb) has a high cytotoxic effect [11, 12], which is reversed in the presence of a muscarinic antagonist and absent in non-tumor breast cells. This selective effect may be due to the fact that murine and human mammary tumors express muscarinic acetylcholine receptors (mAChRs) while normal breast tissue does not [11, 12]. This suggests that mAChRs are potential candidates for drug action in cancer treatment [12–14].

Besides, it is known that the tumor response to treatment may be modulated by many factors, including the presence of environmental pollutants [15, 16]. One of them is hexachlorobenzene (HCB), a persistent organic pollutant that has been used as a fungicide for years until it was banned due to the determination of its several health deleterious effects [17]. However, HCB is accumulated in the soil, water and biota and is still released into the environment as a by-product of chlorination [18]. Furthermore, given its lipid-soluble characteristic, HCB tends to accumulate in fatty tissue like mammary glands [19], and its presence has been detected in different human samples [20–23]. HCB behaves as an endocrine disruptor since it interferes with the synthesis, metabolism, and action of different hormones [24]. It is also related to the promotion of carcinogenesis, angiogenesis and metastasis in hormone-dependent breast cancer [25, 26] and is associated with the increase in tumor cell migration and invasion in TNBC [27, 28].

HCB is an organochlorine compound that acts as a weak aryl hydrocarbon receptor (AhR) agonist, which is a cytosolic member of the basic-helix-loop-helix Per-ARNT-Sim superfamily of transcription factors [29]. When activated by endogenous or exogenous agonists, it translocates to the cell nucleus and activates the expression of several genes that regulate different physiological and pathological functions. The pathological activation of AhR has been related to the development of different diseases, including cancer [30]. Particularly, in breast cancer, AhR is overexpressed compared to normal tissue [31] and its activation is associated with an increased expression of drug transporters producing drug resistance [32]. In this sense, a previous study has shown that, in breast cancer, the activation of AhR can modulate the response to antitumor treatment with the antibiotic doxorubicin by an increase in the protein expression of cytochrome P450 (CYP) 2D6, CYP2C8 and CYP3A4 [33], but there is lack of evidence about a similar effect of HCB on PX antitumor treatment in TNBC.

Thus, the aim of the present study was to investigate whether the exposure to HCB differentially modulates the efficacy of conventional PX treatment and the metronomic combination of PX and Carb in TNBC cells.

## Materials and methods

### Cell culture

The human TNBC cell lines MDA-MB231 (CRM-HTB-26, basal B) and MDA-MB468 (HTB-132, basal A) were obtained from the American Type Culture Collection (ATCC; Manassas, Virginia, USA) and cultured in plastic flasks in DMEM:F12 (1:1) medium [Invitrogen Incorporation (Inc.), Carlsbad, California, USA] with 80 μg/mL gentamicin and 2 mmol/L L-glutamine, supplemented with 10% heat-inactivated fetal bovine serum (FBS; Internegocios anonymous company, Mercedes, Buenos Aires, Argentina). Cells were maintained at 37℃ in a humidified atmosphere containing 5% CO_2_. The medium was changed three times a week. Cells were then detached to be amplified or to develop the experiments using Ca^2+^- and Mg^2+^-free phosphate buffer saline (PBS) containing 0.25% trypsin and 0.02% ethylenediaminetetraacetic acid (EDTA). The absence of mycoplasma was observed by Hoechst staining [34]. Briefly, cells were monthly seeded on slides. After obtaining 40–50% of confluence, cells were fixed with Carnoy’s reagent for 5 min. Then, the fixative was removed and Carnoy’s reagent was added again for 10 min. The slides were then allowed to air dry and stained at room temperature in the darkness with 0.001% bisbenzimide (Hoechst 33258) for 30 min. The samples were then washed with distilled water and air dried. The slides were examined under ultra violet light irradiation (365 nm) and the presence of mycoplasmas was determined as small stippling or bright extracellular rods [34].

In all assays, only cells with a low culture passage (less than 16 since acquisition from ATCC) were used, to avoid genetic drift.

### Cell viability assays

**Figure 1:**
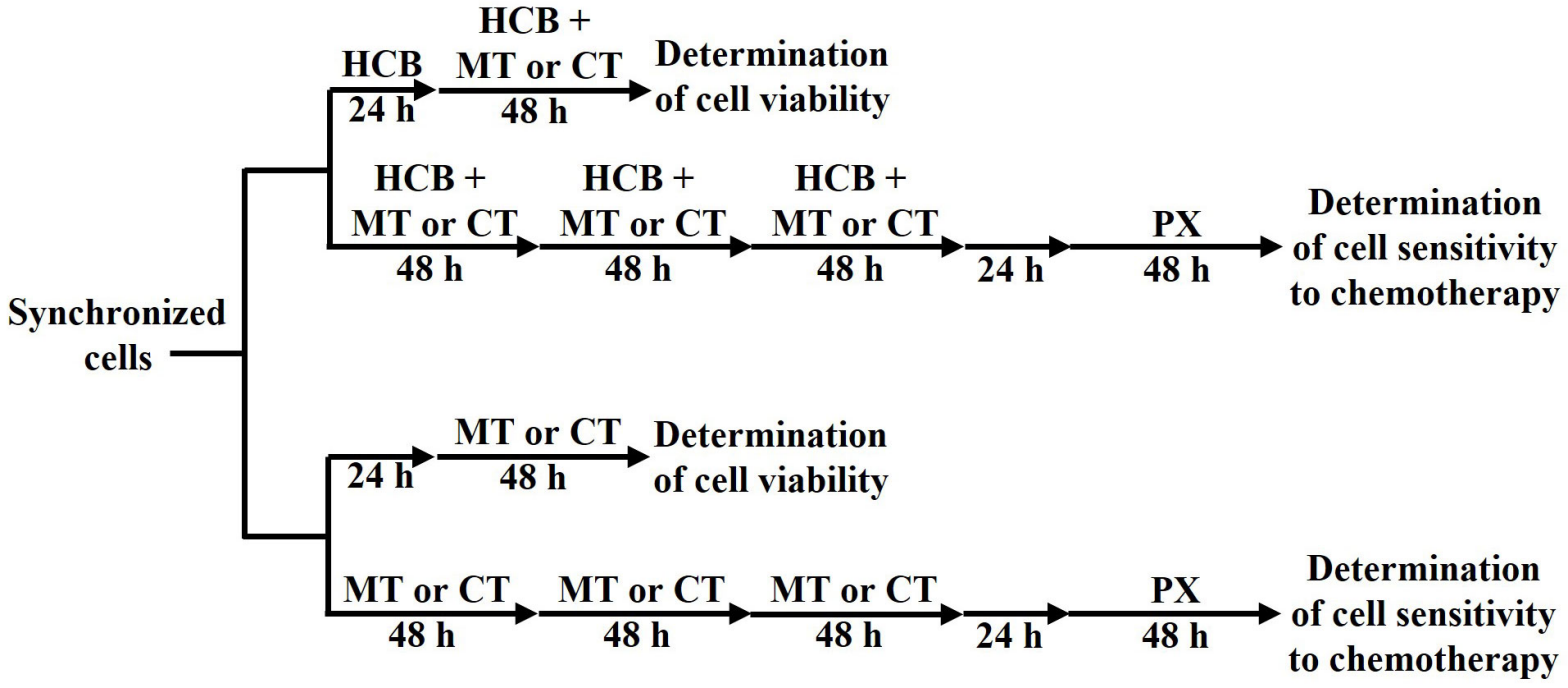
Treatment administration schemes applied to determine cell viability or cell sensitivity to chemotherapy. CT: conventional therapy

Cell viability after treatment was determined by the 3-(4,5-dimethylthiazol-2-yl)-2,5-diphenyltetrazolium bromide (MTT) staining method (Life Technologies, Eugene, Oregon, USA). Briefly, 15,000 cells/well were seeded in 96-well plates in culture medium supplemented with 10% FBS and then left to attach overnight. Then, cells were deprived of FBS for 24 h to induce the synchronization of cultures and pre-treated or not with HCB (> 99% purity, Sigma-Aldrich, Steinheim, Baden-Württemberg, Germany; 1 pmol/L to 1 mmol/L) for 7 days. In the last 2 days, cells were also treated or not with PX (Bristol-Myers Squibb, Buenos Aires, Argentina) in a conventional therapeutic concentration (PX ther; 100 nmol/L) or with a metronomic combination of PX (10 nmol/L) plus Carb (10 pmol/L). To determine the participation of AhR and the NF-κB pathway in the effects of the treatments, cells were previously treated with the AhR inhibitor α-naphthoflavone (NPF; Sigma-Aldrich, Saint Louis, Missouri, USA, 1 µmol/L) or with the inhibitor of NF-κB kinase subunit beta (IKKβ) *N*-(3,5-bis-trifluoromethyl-phenyl)-5-chloro-2-hydroxy-benzamide (IMD; Sigma-Aldrich, St. Louis, MO, USA, 500 nmol/L), which blocks NF-κB inhibitor alpha (IκBα) phosphorylation that in turn blocks the NF-κB pathway. After treatments, the culture medium was replaced by 110 μL of MTT solution (10 μL of 5 mg/mL MTT in PBS plus 100 μL medium free of phenol red) in each well. After incubation for 4 h at 37℃, formazan production from MTT was measured by determining the absorbance at 540 nm with an ELISA reader (BioTek, Winooski, Vertmont, USA; Figure 1).

Then, the modulation of cell sensitivity to PX exerted by two cycles of PX ther or by three cycles of the metronomic combination of PX plus Carb was analyzed, in the absence or presence of HCB pre-treatment. Briefly, 100,000 cells were seeded in flasks containing 10% FBS-supplemented medium and left to adhere overnight. Then, cells were deprived of FBS for 24 h to induce the synchronization of cultures. Cells were pre-treated or not with HCB (10 nmol/L) for 10 days. In the last 6 days, cells were also treated or not with two 48-h cycles of conventional PX ther (100 nmol/L) with a 48-h drug-free interval or with three 48-h cycles of the metronomic combination of PX (10 nmol/L) plus Carb (10 pmol/L). After that, cells were detached using PBS containing 0.25% trypsin and 0.02% EDTA and seeded in 96-well plates. Cells were left to adhere overnight and then deprived of FBS for 24 h to induce the synchronization of cultures. Next, cells were treated with PX (1 pmol/L to 1 mmol/L) and cell viability was determined by the MTT assay (Figure 1).

Values are presented as percentage of cell viability in comparison to cells without treatment (C): considered as 100%, and values are expressed as mean ± standard error of the mean (SEM).

### Trypan blue exclusion assay

Cell viability was also determined by the trypan blue dye exclusion test. Briefly, cells were treated in the same manner as described in the cell viability assays. Then, cells were collected and centrifuged at 850 rpm for 10 min and pellets were resuspended in medium:trypan blue solution (1:1 ratio). The number of viable cells, identified as non-stained cells, was determined using a hemocytometer under an inverted microscope at 10× magnification. Values are presented as the percentage of viable cells with respect to the total cells and values are expressed as mean ± SEM.

### Effective concentration 50 calculation

The effective concentration 50 (EC50) for the different treatments was calculated using the GraphPad Prism6 software. Data with less than 20% in the coefficient of variation for EC50 values were considered appropriate.

### Detection of ABCG2 expression by western blot

Cells (3 × 10^6^) were washed twice with PBS and lysed in 0.3 mL of a buffer composed of 50 mmol/L Tris-HCl, 50 mmol/L NaCl, 1 mmol/L NaF, 1 mmol/L MgCl_2_, 1 mmol/L EDTA, 1 mmol/L ethylene glycol-bis(2-aminoethylether)-N,N,N',N'-tetraacetic acid, 5 mmol/L phenylmethanesulfonyl fluoride, 1% Triton X-100 and 5 μg/mL trypsin inhibitor, aprotinin and leupeptin, pH 7.4. Then, lysates were left in an ice bath for 60 min and later centrifuged at 750 *g* for 10 min at 4℃. Supernatants were saved at −80℃ and protein concentration was analyzed by the Bradford assay [35]. Samples (70 μg protein per lane) were subjected to 12% sodium dodecyl sulfate-polyacrylamide gel electrophoresis minigel electrophoresis, transferred to nitrocellulose membranes, and incubated overnight with rabbit anti-human ABCG2 polyclonal antibodies (Santa Cruz Biotechnology Inc., Dallas, Texas, USA) diluted 1:200. Then, strips were incubated with anti-rabbit immunoglobulin G coupled to horseradish peroxidase diluted 1:10,000 in 20 mmol/L Tris-HCl buffer, 150 mmol/L NaCl and 0.05% Tween 20 at 37℃ for 1 h. Bands were visualized by chemiluminescence. The results of densitometric analysis are expressed as optical density units relative to the expression of glyceraldehyde 3-phosphate dehydrogenase (GAPDH; Santa Cruz Biotechnology Inc., San Diego, CA, USA), which was used as loading control [36].

### Statistical analysis

The significance of the differences between mean values in all control and test samples was evaluated by the GraphPad Prism6 computer program, applying one-way ANOVA followed by Tukey test post-hoc analysis. Differences were considered significant if *P* < 0.05. Results are expressed as mean ± SEM. Previously, it was verified that the data obtained meet the assumptions of normal distribution and homogeneity of variance by the Shapiro-Wilks and Levene tests respectively.

### Drugs

All drugs used for the assays were purchased from Sigma Chemical Company (Saint Louis, Missouri, USA), unless otherwise indicated.

## Results

### Effect of HCB on MDA-MB231 cell viability

**Figure 2:**
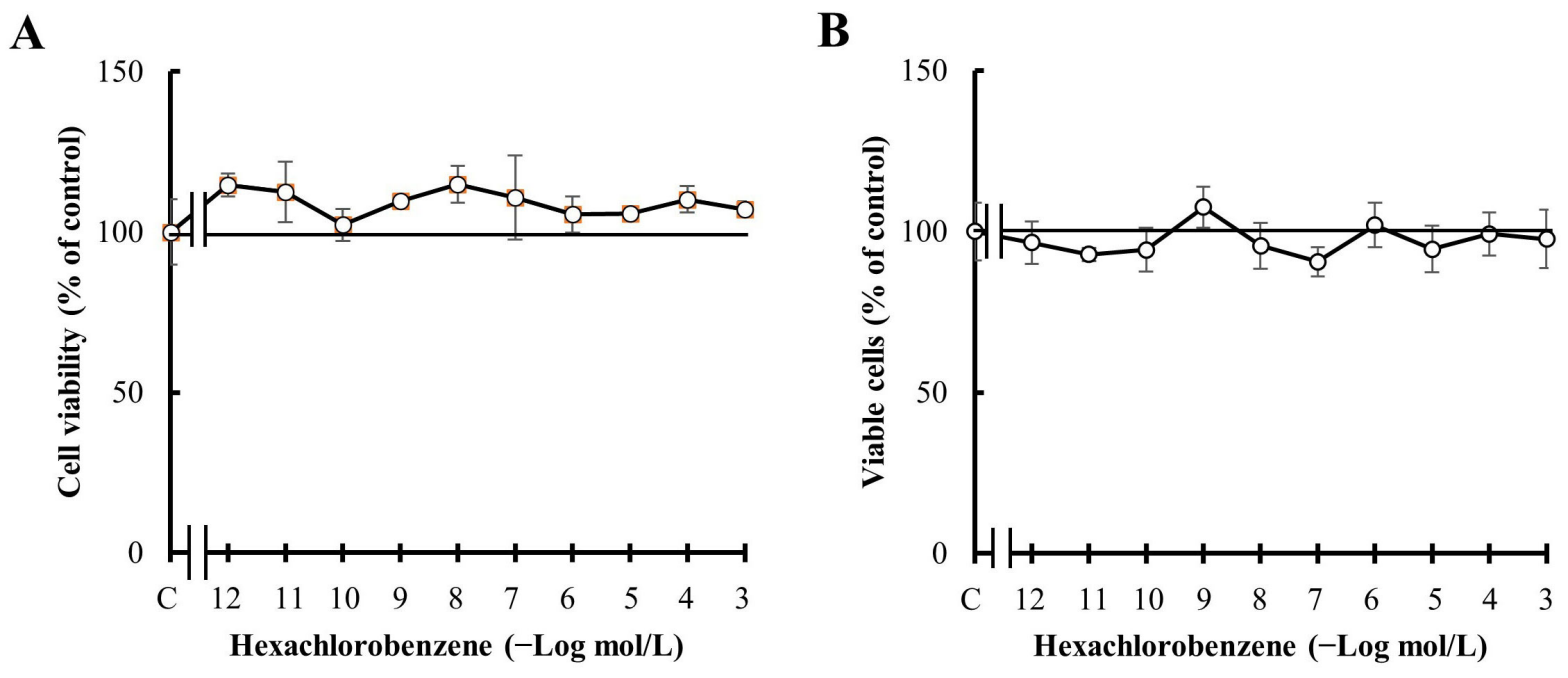
Effect of HCB treatment. (A) Concentration-response curve of HCB on MDA-MB231 cell viability; (B) concentration-response curve of HCB on percentage of MDA-MB231 viable cells. Values are mean ± SEM of four experiments performed in duplicate

First, the effect of HCB administered for 7 days on MDA-MB231 cells was analyzed, since several authors have demonstrated that this treatment time is enough to determine effects *in vitro* similar to those observed *in vivo* [37, 38]. The results demonstrated that the addition of HCB at concentrations from 1 pmol/L to 1 mmol/L did not modify cell viability (Figure 2A). To confirm the absence of effect of HCB on cell viability, the percentage of living cells was analyzed by using the trypan blue exclusion assay after the different treatments. The results represented in Figure 2B show values similar to those obtained by the MTT assay (Figure 2A).

### Conventional and metronomic chemotherapy treatment of cells in the presence of HCB

Since previous reports have shown that AhR is functional in MDA-MB231 cells [39], studies were next performed to determine whether the effects of conventional and metronomic antitumor therapies were modulated in the presence of HCB.

**Figure 3:**
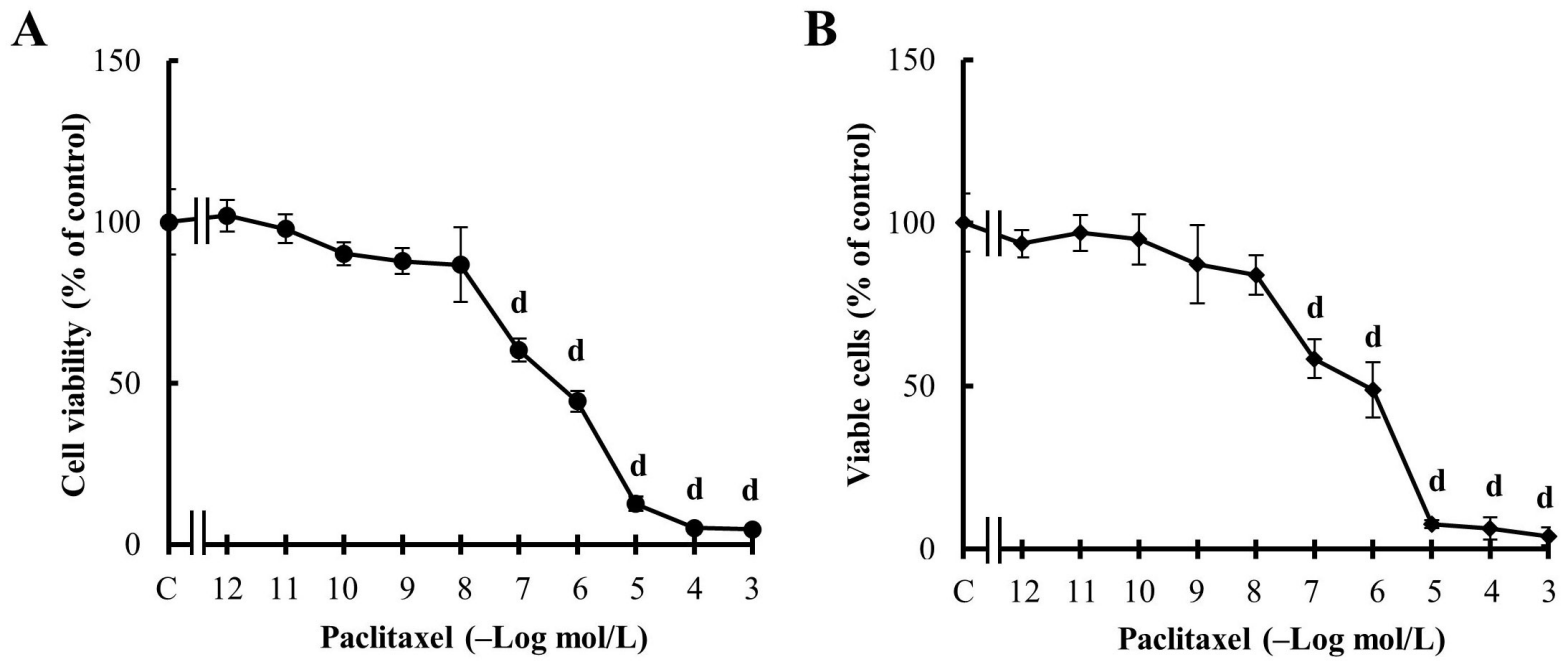
Effect of PX treatment. (A) Concentration-response curves of PX on cell viability; (B) concentration-response curves of PX on viable cell percentage. Values are mean ± SEM of four experiments performed in duplicate. d: *P* < 0.001 *vs.* C: considered as 100%

First, the results confirmed that PX reduced MDA-MB231 cell viability in a concentration-dependent manner and that PX effect was significant at concentrations equal to or higher than 100 nmol/L (Figure 3A), similar to previous results [40]. The effect of PX treatment was confirmed by analyzing the percentage of living cells by the trypan blue exclusion assay. The results, presented in Figure 3B, show values similar to those obtained by the MTT assay shown in Figure 3A.

Previous results determined that the combination of PX plus Carb in a metronomic administration scheme (PX: 10 nmol/L + Carb: 10 pmol/L) is effective in reducing the viability of MDA-MB231 cells with a potency similar to CT [40]. Therefore, here the objective was to determine whether the response to conventional or MT can be modulated by HCB administered for 7 days.

**Figure 4:**
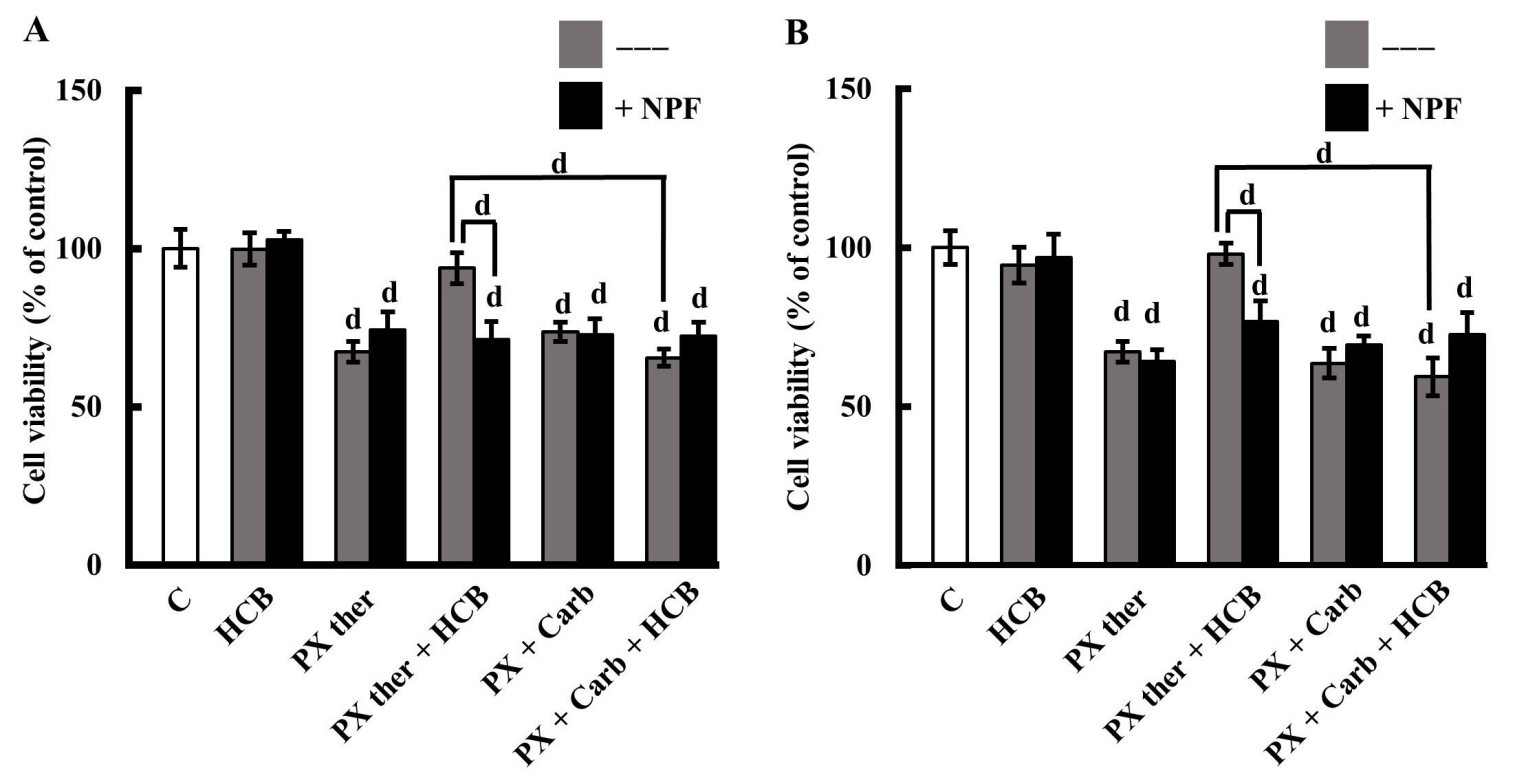
Role of AhR in HCB effects on conventional and metronomic treatments. (A) Viability of MDA-MB231 cells treated with PX in a conventional therapeutic concentration (PX ther; 100 nmol/L) or with the metronomic combination of PX + Carb, alone or in combination with HCB (10 nmol/L), in the absence (grey columns) or presence (black columns) of the AhR inhibitor NPF; (B) viability of MDA-MB468 cells treated with PX in a conventional therapeutic concentration (PX ther; 100 nmol/L) or with the metronomic combination of PX + Carb, alone or in combination with HCB (10 nmol/L), in the absence (grey columns) or presence (black columns) of the AhR inhibitor. Values are the mean ± SEM of four experiments performed in duplicate. d: *P* < 0.0001 *vs.* C: considered as 100%

The results confirmed that PX ther (100 nmol/L) reduces the viability of these cells with a potency similar to the metronomic combination of PX + Carb (PX ther: 67.27% ± 3.22%; PX + Carb: 73.66% ± 3.03%). They also showed that the pre-treatment of cells with an environmental concentration of HCB (10 nmol/L) modified the cell response to the PX ther treatment to values similar to C (93.74% ± 4.94%) and that the presence of HCB did not modify the response to the metronomic chemotherapy combination (65.43% ± 2.77%; Figure 4). Then was evaluated whether the HCB modulation of the effect of PX ther on cell viability was mediated by AhR activation, since pre-incubation with the AhR inhibitor NPF reversed cell viability to values close to those determined for PX ther alone (HCB + PX ther + NPF: 71.30% ± 5.64%; Figure 4A).

Moreover, these treatments in other TNBC were studied cells to determine whether the metronomic combination in the presence of HCB maintains its effectiveness. In MDA-MB468 cells derived from another subtype of TNBC, previous results described that the metronomic administration of PX plus Carb (PX: 1 nmol/L + Carb: 0.1 nmol/L) is effective in reducing the cell viability [40]. Here, the results described that HCB did not modify cell viability. PX ther was determined to reduce cell viability in a manner similar to the metronomic combination of PX + Carb (PX ther: 67.27 ± 3.22%; PX + Carb: 63.61% ± 4.61%). The results described that although HCB (10 nmol/L) did not modify cell viability, it reduced the cell response to the PX ther treatment to values near to those of C (97.98% ± 3.36%), without affecting the response to the metronomic chemotherapy combination (59.38% ± 5.95%). Similar to that observed in MDA-MB231 cells (Figure 4A), only the effect of HCB plus PX ther on cell viability was mediated by AhR (HCB + PX ther + NPF: 76.74% ± 6.4%; Figure 4B).

**Figure 5:**
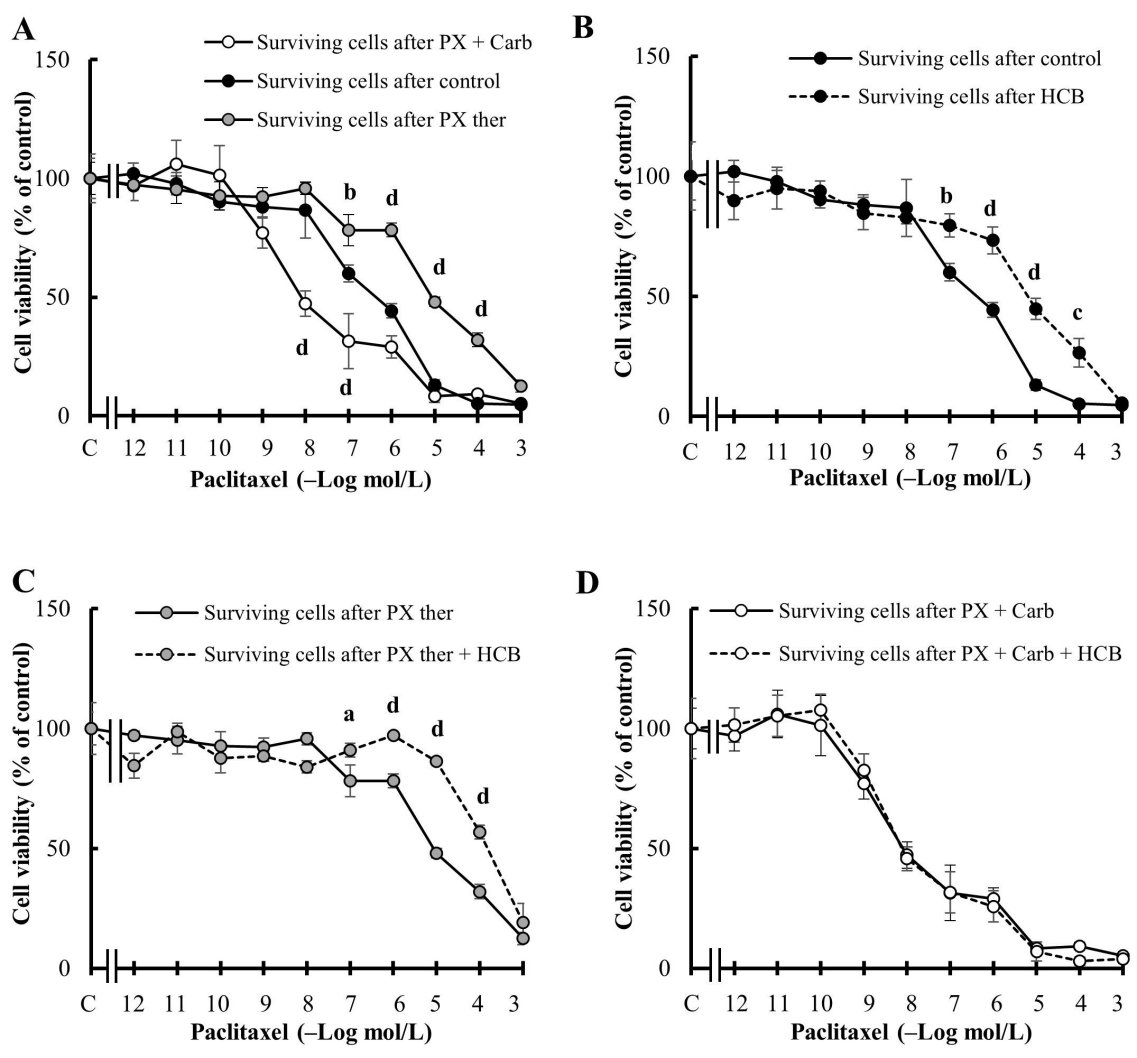
Sensitivity of MDA-MB231 cells to chemotherapy. (A) Concentration-response curves of PX on the viability of surviving cells after two cycles of PX in a conventional therapeutic concentration (PX ther) treatment (100 nmol/L) or after three cycles of the metronomic combination of PX + Carb; (B) effect of HCB (10 nmol/L) on the concentration-response curves of PX, on the viability of surviving cells without another treatment; (C) effect of HCB (10 nmol/L) on the concentration-response curves of PX, on the viability of surviving cells after two cycles of PX ther treatment (100 nmol/L); (D) effect of HCB (10 nmol/L) on the concentration-response curves of PX, on the viability of surviving cells after three cycles of the metronomic combination of PX + Carb. Values are mean ± SEM of four experiments performed in duplicate. a: *P* < 0.05; b: *P* < 0.01; c: *P <* 0.001; d: *P* < 0.0001 *vs.* (A) without treatment surviving cells or (B) and (C) surviving cells in the absence of HCB

Then, the modulation of the sensitivity to PX chemotherapy of the MDA-MB231 cells surviving the different treatments was studied. Cells surviving the PX ther treatment were less sensitive to a subsequent PX cycle than C (EC50 control: 213.10 nmol/L, EC50 PX ther: 5.82 µmol/L, *P* = 0.00340), while the cells surviving the metronomic combination of PX + Carb were more sensitive to the last PX cycle (EC50: 9.34 nmol/L, *P* = 0.00691 *vs.* EC50 control; Figure 5A). In the presence of HCB, surviving cells decreased their sensitivity to the last PX cycle (EC50: 5.75 µmol/L, *P* = 0.00356 *vs.* EC50 control) with respect to C (Figure 5B). This tendency towards a decrease in cell sensitivity to a subsequent PX cycle caused by HCB was also observed in the cells surviving the treatment with PX ther in the presence of HCB (EC50: 76.59 µmol/L; Figure 5C). When assays to evaluate the response of cells surviving the treatment with the metronomic combination to another cycle with PX were made no significant differences in cell viability between cells pre-treated or not with HCB were observed (EC50: 10.57 nmol/L and EC50: 9.34 nmol/L respectively; Figure 5D).

### Mechanism involved in the effect of HCB on the response of tumor cells to conventional or metronomic treatment

A relevant aspect in cancer chemotherapy is the development of resistance to treatment. In this regards, previous reports have indicated that activation of the NF-κB pathway induces the expression of proteins that can modulate cell sensitivity to chemotherapy [41]. Given that the treatments with HCB or conventional and metronomic therapies modulated the resistance to chemotherapy in cells that survived the treatments (Figure 5), the participation of the NF-κB pathway on cell viability was next evaluated.

**Figure 6:**
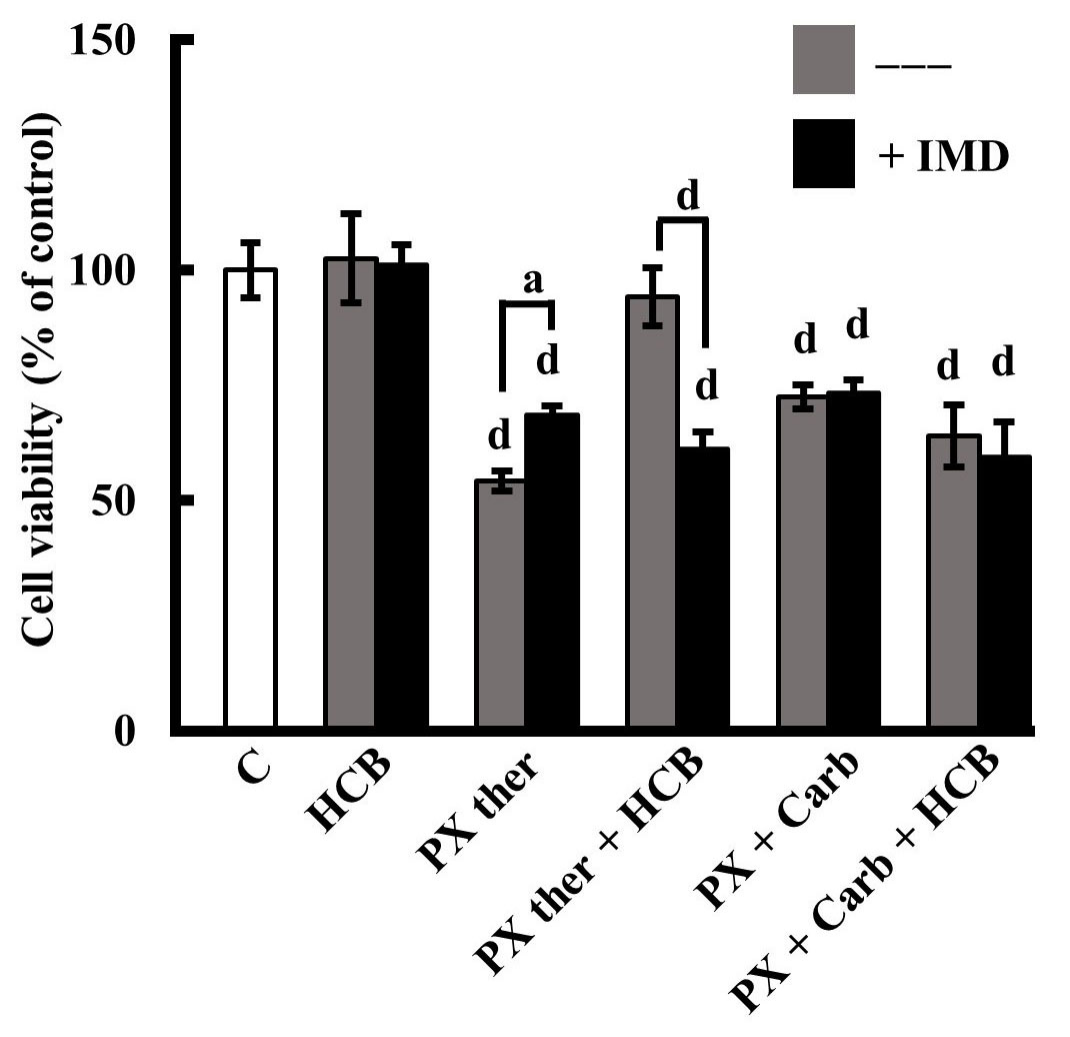
Role of NF-κB in the effects of PX and HCB on conventional and metronomic treatments. Determination of viability in MDA-MB231 cells treated with PX in a conventional therapeutic concentration (PX ther; 100 nmol/L) or with the metronomic combination with PX (10 nmol/L) + Carb (10 pmol/L), alone or in combination with HCB (10 nmol/L), in the absence (grey columns) or presence (black columns) of the NF-κB pathway inhibitor IMD. Values are the mean ± SEM of four experiments performed in duplicate. a: *P* < 0.05; d: *P* < 0.0001 *vs.* C: considered as 100%

Results in Figure 6 show that PX ther partially exerts its biological effects by the NF-κB pathway since pre-treatment of cells with the inhibitor of this pathway, IMD, reduced PX ther effect on cell viability (PX ther: 54.25% ± 2.22%; PX ther + IMD: 68.50% ± 2.08%, *P* < 0.05). The results also determined that the action of HCB on the response to the PX ther treatment (94.25% ± 6.29%) was mediated by the activation of the NF-κB pathway since the pre-treatment of the cells with IMD reversed the HCB effect to a value similar to that of PX ther alone (HCB + IMD + PX ther: 61.25% ± 3.59%). Besides the results determined that the NF-κB pathway was not involved in the effect of the treatment with the metronomic combination in the absence or presence of HCB (both *P* > 0.05), indicating that the signaling pathways involved in the modulation of cell viability in the MT and in the CT are different (Figure 6). The results also confirmed that IMD alone did not modify cell viability (data not shown).

Since the results determined the participation of the NF-κB pathway in the effects of PX ther and HCB (Figure 6) and the modulation of chemotherapy response by HCB in surviving cells (Figure 5), the participation of the drug extrusion protein ABCG2 in the modulation of sensitivity of the cells surviving the treatments was investigated, because this protein has been linked with the development of chemotherapy resistance.

**Figure 7:**
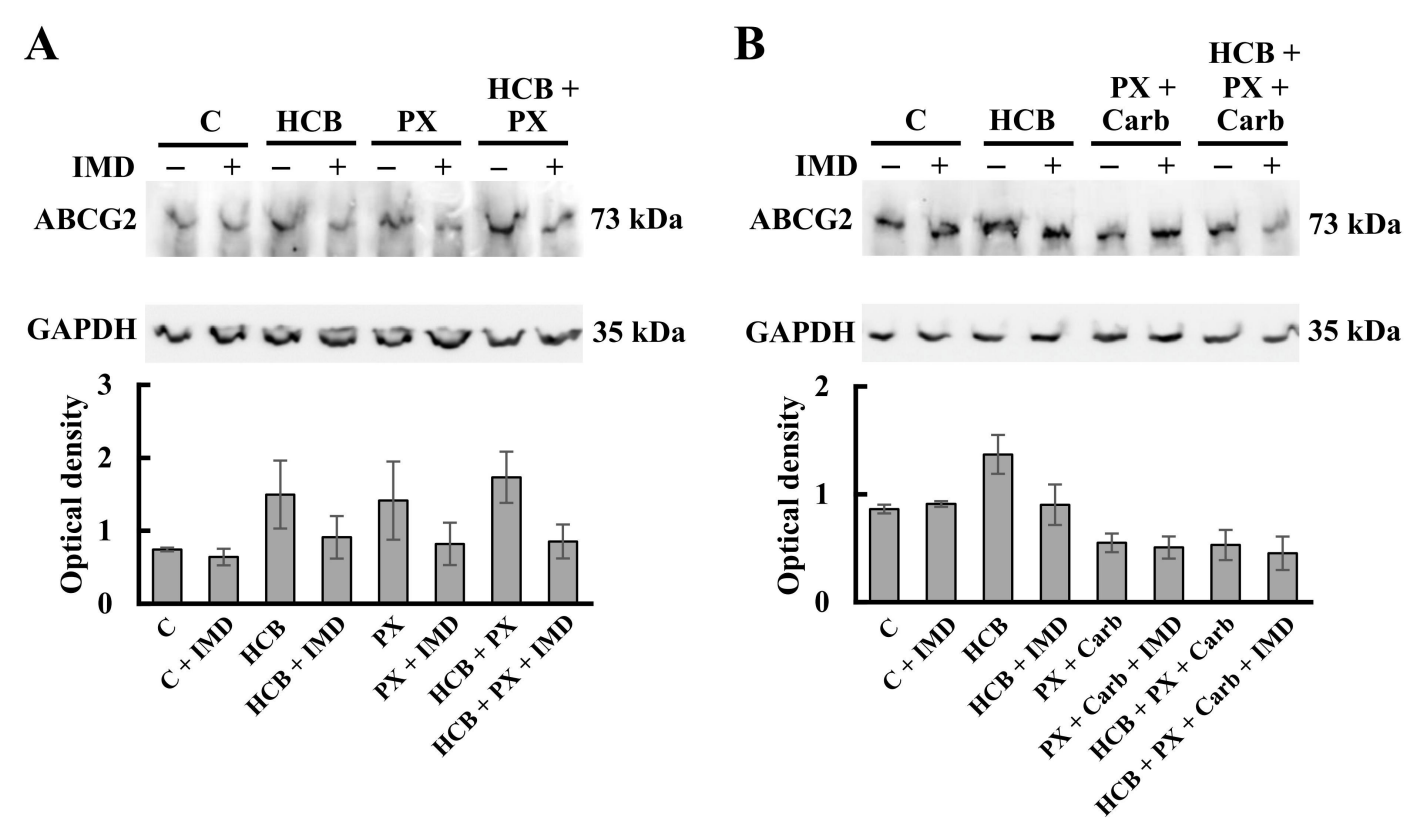
Expression of ABCG2 in MDA-MB231 cells. (A) Expression of ABCG2 determined by western blot assays in cells treated with HCB (10 nmol/L), PX (100 mmol/L) or both, in the absence or presence of the NF-κB pathway inhibitor IMD (500 nmol/L); (B) expression of ABCG2 determined by western blot assays in cells treated with PX: 10 nmol/L + Carb (10 pmol/L), HCB (10 nmol/L) or both, in the absence or presence of IMD (500 nmol/L). Molecular weights are indicated on the right. Densitometric analysis of the bands is expressed as optical density units relative to the expression of GAPDH protein used as loading control. One representative experiment of three is shown. Values are the mean ± SEM of three experiments

The results demonstrated that these cells basally express ABCG2 and that both HCB and PX ther treatments, alone or combined, increased ABCG2 expression, with the participation of the NF-κB pathway since the pre-treatment with IMD decreased these effects (Figure 7A). The results also determined that the treatment of cells with the metronomic combination of PX + Carb decreased ABCG2 protein expression (*P* < 0.05). Although the HCB pre-treatment increased ABCG2 expression, when the cells were then treated with the metronomic combination (*P* < 0.05), no significant differences in the expression of this protein between cells pre-treated or not with HCB were observed, indicating that the MT studied totally reversed the effect of HCB on ABCG2 expression (Figure 7B).

## Discussion

HCB is a persistent organic pollutant currently detected in different human biological samples such as serum and breast milk worldwide [42–44]. Different authors have found a correlation between serum and tissue concentration levels of HCB and the probability to develop cancer [45–48]. Although HCB is known to be a weak AhR agonist, the effect of the activation of this receptor on breast carcinogenesis is still unclear. Some authors have suggested that this activation favors carcinogenesis [49, 50], whereas others have indicated that it prevents this process [51, 52]. In agreement with this dilemma, it has also been proposed that the activation of AhR could modulate the response of tumor cells to antitumor chemotherapy in breast cancer either positively [53] or negatively [54]. Thus, the present study aimed to investigate the ability of HCB to modulate the response of TNBC MDA-MB231 cells to conventional chemotherapy as well as the probable benefits of replacing it by MT.

Some authors have described that AhR activation can modulate the cell cycle [55] by increasing cell proliferation [56] and tumor growth in mice [57]. Since MDA-MB231 cells express functional AhR [37], the effect of the administration of HCB on cell viability was determined. The results described that HCB did not modify cell viability at any of the concentrations tested. These results are related with those obtained by García et al. [58], who described that HCB did not modify MDA-MB231 cell proliferation at a concentration range from 5 µmol/L to 5 nmol/L.

Previous reports have indicated that TNBC patients benefit from the administration of PX in adjuvant therapy, supporting the idea that taxanes as PX are useful for this group of patients [59, 60]. Here, the results confirmed that MDA-MB231 cells respond to the treatment with PX administered either in a conventional schedule or in a metronomic combination with Carb, since cell viability and proliferation were reduced in a dose-dependent manner (Figure 3). These results are in accordance with previous ones described [40].

In the present study, when HCB was administered alone, it did not modify cell viability or proliferation. However, several authors have described that AhR activation reduces the sensitivity to different antitumor therapies [61–63]. Specifically concerning chemotherapy, Goode et al. [64] established the importance of AhR as modulators of the response, since these authors described that the knockout of this receptor in human breast tumor cells increased the sensitivity to a subsequent PX treatment. However, there is no information about the effect of the activation of AhR by organochlorines as HCB on the response to PX, either in a conventional or a metronomic administration schedule.

Here, the results described that, in MDA-MB231 cells, HCB reduces the response to a conventional schedule of PX treatment and that this effect is mediated by the activation of AhR, since it was reversed by NPF (Figure 4). This HCB modulation is similar to that observed by Hsieh et al. [33], who determined that the treatment of human breast cancer cells with di(2-ethylhexyl)phthalate, an environmental pollutant that is a weak agonist of AhR, reduces the antitumor response to doxorubicin due to the increase in CYP3A4 and CYP2C8 expression, which in turn increases the metabolization rate of the drug. This signaling/metabolization pathway could also be involved in the HCB modulation of the response to PX since these enzymes of the CYP family also play an important role in the metabolism of PX and it is known that an increase in their expression or activity reduces the PX half-life, thus decreasing chemotherapy efficacy in cancer patients [65].

Another mechanism by which HCB exposure could reduce the efficacy of conventional PX treatment is mediated by an increase in the expression levels of the PX extruder pump ABCG2. Regarding this, Chung et al. [66] described that the activation of AhR can increase ABCG2 expression in ovarian tumor cells, which in turn would reduce the action time and efficacy of PX. These results highlight the relevance of the participation of AhR in the modulation of the sensitivity to PX treatment because, for the first time, an environmental concentration of HCB was demonstrated to exert a modulatory effect on the response to the antitumor PX treatment administered in a conventional therapeutic concentration by the activation of AhR.

Previous results described that PX causes similar effects on tumor and normal mammary cells, which is clearly undesirable [40]. This is one of the reasons why specific adjuvant regimens for TNBC are still under review and third-generation chemotherapy regimens using dose-dense or metronomic polychemotherapy are thought to be more effective and less harmful than traditional chemotherapy. Considering this fact and that previous results have demonstrated that MDA-MB231 cells are sensitive to a therapy based on a metronomic combination of PX plus muscarinic agonists [40], the study whether HCB modulates the response to this metronomic chemotherapy was decided.

The results determined that MT resulted as effective as CT and that the response was not modified by pre-treatment with HCB. This could be due to the differences in the mechanism of action of both therapies. On one hand, in CT, PX must reach the cell cytoplasm to exert its stabilizing effect on microtubules [67, 68], so it can be extruded by ABCG2 proteins [69, 70] or metabolized by CYP proteins [65]. On the other hand, when PX and Carb are administered in a metronomic scheme, they exert a pro-apoptotic effect by the activation of mAchRs, which are present in the cell membrane [12]. So, the efficacy of this scheme would not be affected by the expression levels of ABCG2 or CYP.

A method to study whether a treatment modifies the sensitivity to chemotherapy is to administer successive cycles of the treatment and then determine the sensitivity to chemotherapy. Previous reports have indicated that the treatment of oncologic patients with PX may lead to the reduction of chemotherapy efficacy, causing an effect of acquired resistance [71–74]. The present results confirmed that successive cycles of PX treatment induce a significant decrease in cell sensitivity to a subsequent PX administration and that this effect is potentiated by the HCB pre-treatment, probably due to an addition of effects. These results are in agreement with those obtained by Zhao et al. [74] and Jeong et al. [75], who determined the appearance of acquired resistance in TNBC after successive taxane cycles.

In contrast, the administration of successive cycles of the metronomic combination treatment induced an increase in cell sensitivity to PX. These results are in accordance with others previously published, where the MT based on PX combined with muscarinic agonists induced a decrease in ABCG2 expression and the consequent increase in cell sensitivity to a subsequent PX treatment, due to the increase in the time during which PX remained in the intracellular space exerting its effect [40, 76]. Furthermore, the HCB pre-treatment did not modify the cell sensitivity to PX after the MT, indicating that the effects exerted by HCB on the cells were reversed by the action of this therapy.

Given that the NF-κB pathway can be activated by AhR [77] and is also associated with decreased effectiveness of chemotherapy treatment [78, 79], the participation of this pathway in the effect observed was also studied. The results determined that the effect of PX CT on cell viability is at least partially mediated by the NF-κB pathway in the absence or presence of HCB pre-treatment. These results are in agreement with those obtained by Esparza-López et al. [80] and Kim et al. [6], who described that the treatment with cytostatic drugs activates the NF-κB pathway in breast cancer cells. It is known that, although the conventional PX therapeutic concentration exerts its antitumor effect mainly by stabilizing the cytoskeleton [81], it also activates signaling pathways such as NF-κB [82]. In turn, this increases the expression of MRPs, which reduces sensitivity to PX and stimulates tumor progression [83].

Resistance to treatment, which can be innate or acquired, is one of the most important undesirable effects of antitumor therapy. PX can induce acquired resistance [84, 85] by the transactivation of signaling pathways [86] or by the induction of overexpression of several proteins [87, 88]. One of the proteins involved in the resistance to PX treatment in breast adenocarcinomas is the ABCG2 pump transporter [89]. Although this PX-mediated increase in ABCG2 expression has been previously described by Duz et al. [5], the results here demonstrated that HCB or PX conventional chemotherapy treatment increased ABCG2 expression levels and that these effects are NF-κB-dependent, since the pre-treatment with the inhibitor of this pathway reversed them. When cells were treated with HCB and PX ther combined, a significant increase in ABCG2 expression level was determined. The PX plus Carb MT was confirmed reduces the levels of this protein in a way similar to that previously described [40]. Surprisingly, when pre-treating these cells with HCB, the expression of ABCG2 was not affected respect to the MT value, indicating that the MT reversed the effect of HCB on ABCG2 expression.

The results are in accordance with those described by Kuhnert et al. [90], who determined that the activation of AhR by prochloraz, an AhR weak agonist, induced an increase in ABCG2 expression levels in bovine breast gland. This increase in ABCG2 mediated by AhR activation partially explains the reduction of the cytostatic effect of PX in the presence of HCB. These results are in agreement with those obtained by Mukherjee et al. [91] and Amawi et al. [92], who described that PX treatment increases the activation and expression of ABCG2 in different breast cancer cell lines and that this factor could mediate the resistance to treatment [5].

Moreover, these results reveal the possibility of generalizing the usage of this combination of drugs in TNBC cells in the presence of HCB, since it is also effective on MDA-MB468 cells, derived from a different TNBC classified as Basal A [93].

**Figure 8:**
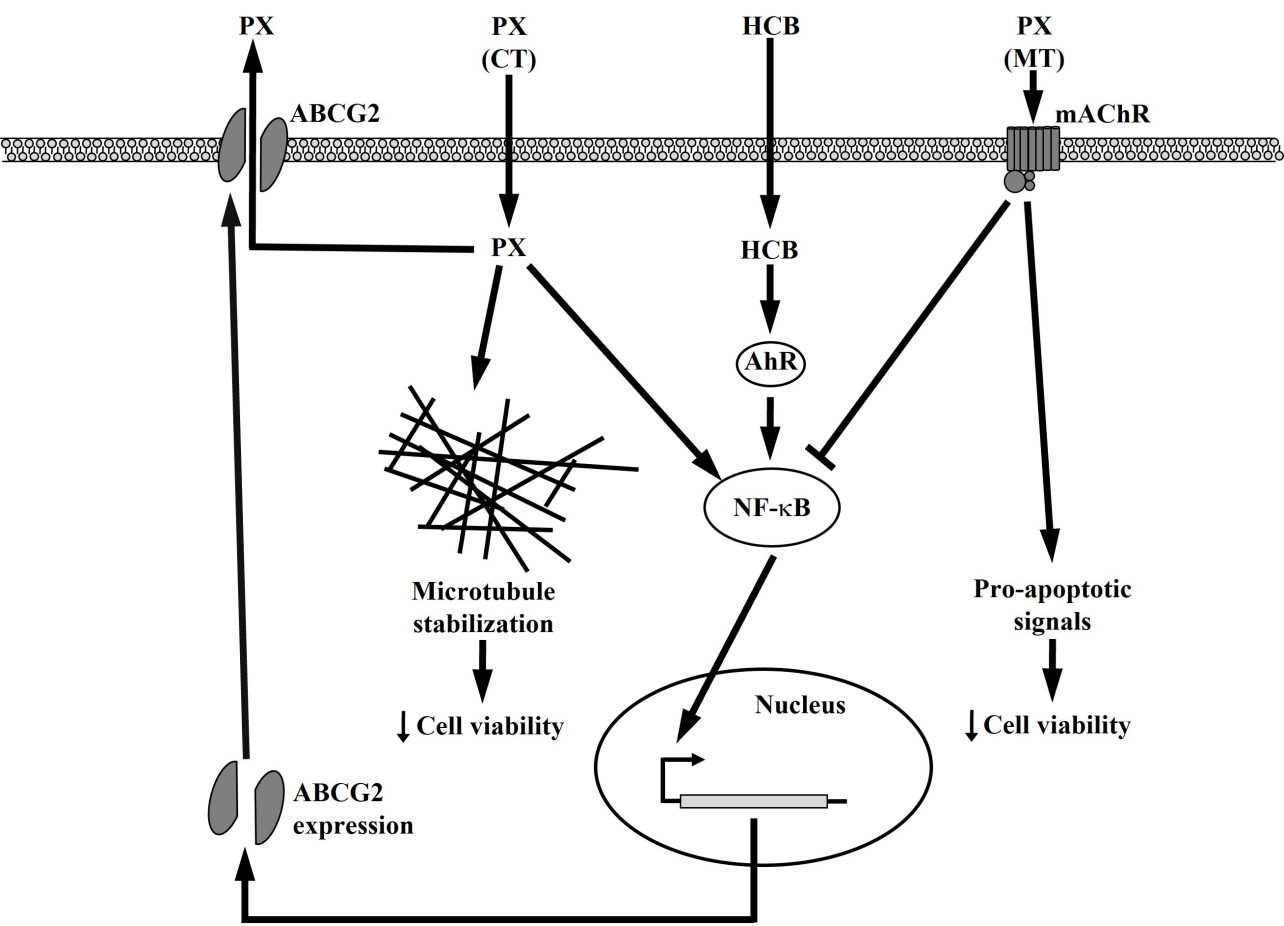
Possible signal transduction pathways in MDA-MB231 cells treated with HCB or conventional and metronomic therapies

A possible mechanism of action of conventional and MT treatments in the presence of HCB in TNBC MDA-MB231 cells is proposed in Figure 8.

In conclusion, the present results allow concluding that either HCB or PX CT increases ABCG2 expression by the activation of the NF-κB pathway, which would leave the surviving cells with a lower sensitivity to a subsequent PX treatment because the high levels of ABCG2 would efficiently extrude it. These effects are even higher in PX treatment with HCB pre-incubation.

Besides, PX plus Carb MT activates mAchR, which induces a decrease in the ABCG2 expression levels by inhibiting the NF-κB pathway, which would sensitize surviving cells to a subsequent PX treatment due to a lower extrusion rate. This would in turn increase the PX time of action in the intracellular space. When pre-treating cells with HCB, the increase in ABCG2 expression is reversed by the MT to levels similar to those obtained with the MT alone, so that the response to a subsequent PX treatment is not affected. This effect would be due to the inhibitory action of the MT on the NF-κB pathway, which would counteract HCB induction of this pathway.

These results indicate that, in TNBC MDA-MB231 cells treated with HCB, the replacement of the PX conventional therapeutic schedule by a metronomic one based on the combination of PX with Carb would be beneficial for antitumor therapy. Although more experiments are needed to extrapolate these conclusions to TNBC patients exposed to HCB, the perspectives are encouraging.

## Abbreviations

ABCG2: ATP-binding cassette transporter G2
AhR: aryl hydrocarbon receptor
Carb: carbachol
CT: conventional therapy
CYP: cytochrome P450
EDTA: ethylenediaminetetraacetic acid
EC50: effective concentration 50
FBS: fetal bovine serum
GAPDH: glyceraldehyde 3-phosphate dehydrogenase
HCB: hexachlorobenzene
IMD: *N*-(3,5-bis-trifluoromethyl-phenyl)-5-chloro-2-hydroxy-benzamide
Inc.: incorporation
mAChRs: muscarinic acetylcholine receptors
MRPs: multidrug resistance proteins
MT: metronomic therapy
MTT: 3-(4,5-dimethylthiazol-2-yl)-2,5-diphenyltetrazolium bromide
NF-κB: nuclear factor kappa B
NPF: α-naphthoflavone
PBS: phosphate buffer saline
PX ther: paclitaxel in a conventional therapeutic concentration
PX: paclitaxel
SEM: standard error of the mean
TNBC: triple negative breast cancer

## Declarations

### Availability of data and materials

Datasets are available on request at aespan_1999@yahoo.com: the raw data supporting the conclusions of this manuscript will be made available by the authors, without under reservation, to any qualified researcher.

### Copyright

© The Author(s) 2024.
